# Supplementary figures and images for: Genetic control of the leaf ionome in pearl millet and correlation with root and agromorphological traits
Source: PLoS One. 2025 May 19;20(5):e0319140. doi: 10.1371/journal.pone.0319140 (PMC12088009; doi:10.1371/journal.pone.0319140)

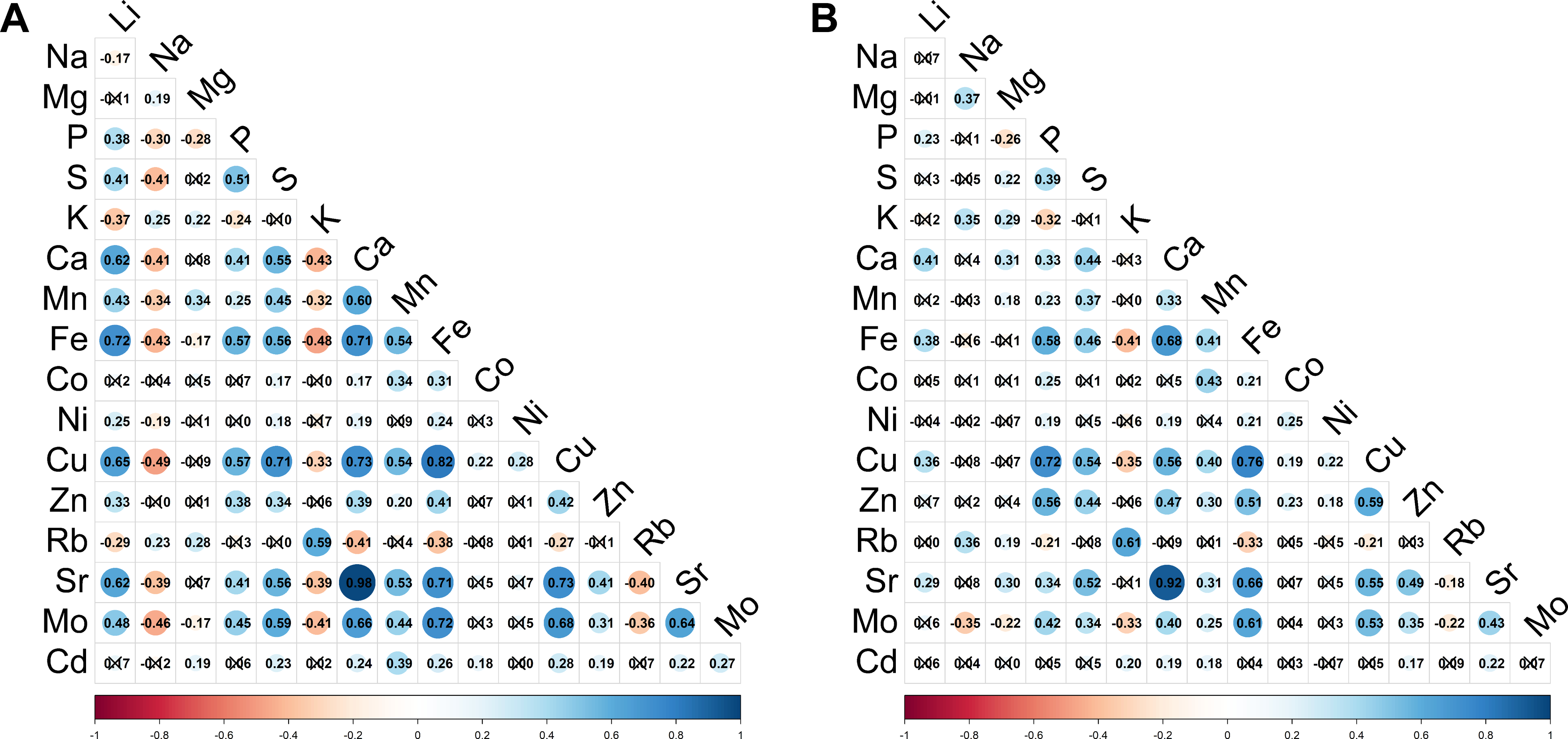

Supplement: S1 File — Field trial experimental design. The design follows a completely randomized block layout with four replicates. Each replicate consists of 10 sub-blocks, each containing 16 plots. Each plot (detail shown in the top right) is planted with three rows of 10 plants of the same genotype, with a spacing of 0.9 m between rows and 0.3 m between plants. The red dots indicate locations where soil samples were collected at 4 different depths (0–140 cm). Leaf samples, soil analyses, and root traits were also studied (illustrations shown at the bottom right). The blue icons represent irrigation pumps. S2 Fig. Correlation plot for soil ion content in 2021 (A) and 2022 (B) field sites. Heatmap representing Pearson’s correlation coefficients between soil ion concentrations measured at four different depths (0–140 cm). Color gradients indicate the Pearson’s correlation coefficient. Non-significant correlations at a p-value threshold of 0.05 are indicated with a cross. S3 Fig. Boxplot representing variation in ion content in 2021 and 2022 in the PMIGAP panel. Ion content is represented as mg/kg. p-values from the Wilcoxon test are represented. S4 Fig. Correlation plot for leaf ion content of the PMIGAP panel measured during the experimental field study. Heatmap representing Pearson’s correlation coefficients between BLUEs of all accessions of the panel observed in 2021 (A) and 2022 (B). Color gradients indicate the Pearson’s correlation coefficient. Non-significant correlations at a p-value threshold of 0.05 are indicated with a cross. S5 Fig. Correlation plot for ion content, root (A) and agro-morphological (B) traits in 2021 and 2022. Heatmap representing Pearson’s correlation coefficients between ion content, root and agro-morphological traits. Color gradients indicate the Pearson’s correlation coefficient. Significant correlations at a p-value threshold of 0.05 are indicated in bold. Roots traits are number of metaxylem vessels (MX_Number), mean area of metaxylem vessels (Meansize [file pone.0319140.s001.zip › Supplementary/S4_Fig.tif]

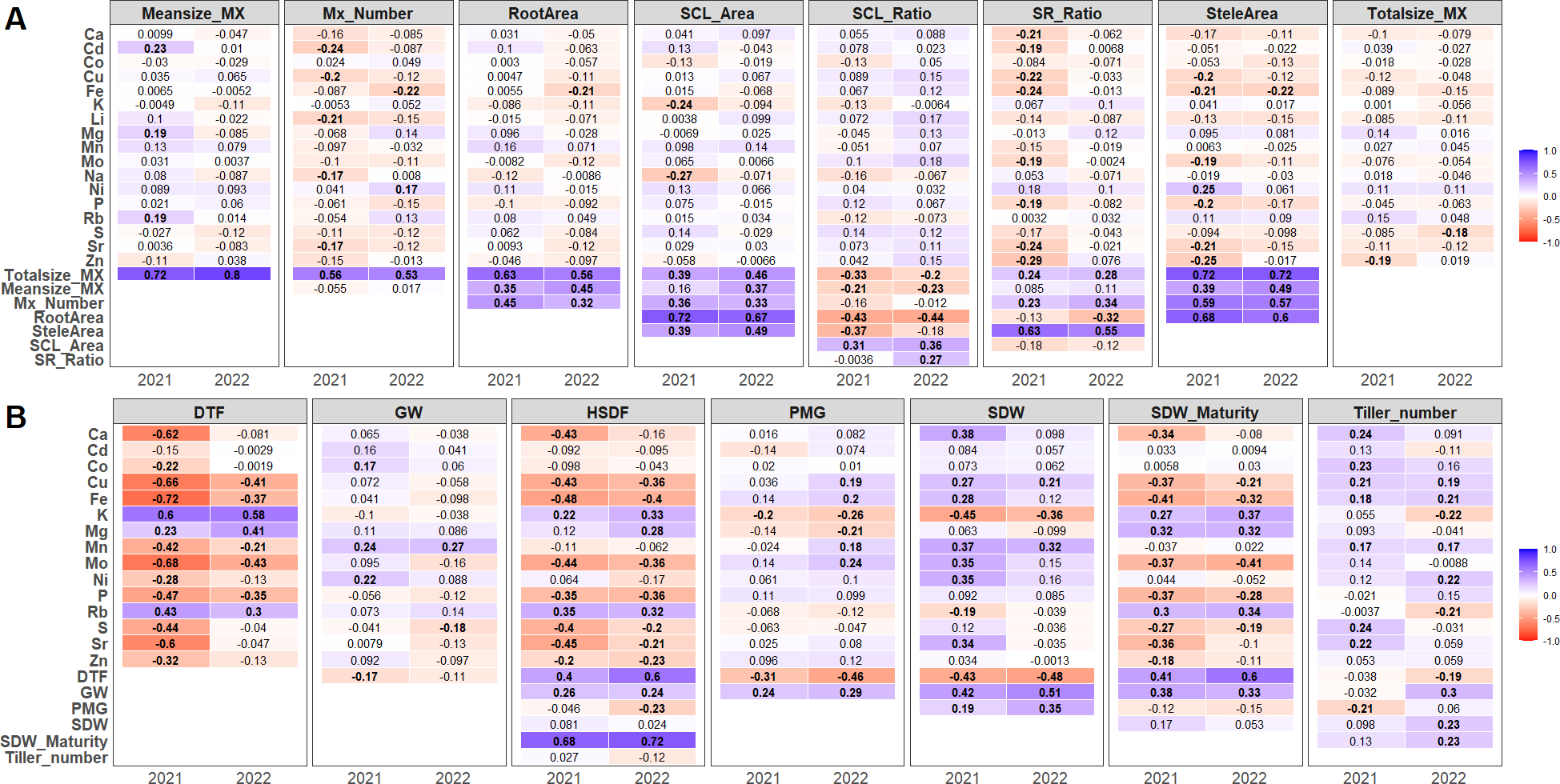

Supplement: S1 File — Field trial experimental design. The design follows a completely randomized block layout with four replicates. Each replicate consists of 10 sub-blocks, each containing 16 plots. Each plot (detail shown in the top right) is planted with three rows of 10 plants of the same genotype, with a spacing of 0.9 m between rows and 0.3 m between plants. The red dots indicate locations where soil samples were collected at 4 different depths (0–140 cm). Leaf samples, soil analyses, and root traits were also studied (illustrations shown at the bottom right). The blue icons represent irrigation pumps. S2 Fig. Correlation plot for soil ion content in 2021 (A) and 2022 (B) field sites. Heatmap representing Pearson’s correlation coefficients between soil ion concentrations measured at four different depths (0–140 cm). Color gradients indicate the Pearson’s correlation coefficient. Non-significant correlations at a p-value threshold of 0.05 are indicated with a cross. S3 Fig. Boxplot representing variation in ion content in 2021 and 2022 in the PMIGAP panel. Ion content is represented as mg/kg. p-values from the Wilcoxon test are represented. S4 Fig. Correlation plot for leaf ion content of the PMIGAP panel measured during the experimental field study. Heatmap representing Pearson’s correlation coefficients between BLUEs of all accessions of the panel observed in 2021 (A) and 2022 (B). Color gradients indicate the Pearson’s correlation coefficient. Non-significant correlations at a p-value threshold of 0.05 are indicated with a cross. S5 Fig. Correlation plot for ion content, root (A) and agro-morphological (B) traits in 2021 and 2022. Heatmap representing Pearson’s correlation coefficients between ion content, root and agro-morphological traits. Color gradients indicate the Pearson’s correlation coefficient. Significant correlations at a p-value threshold of 0.05 are indicated in bold. Roots traits are number of metaxylem vessels (MX_Number), mean area of metaxylem vessels (Meansize [file pone.0319140.s001.zip › Supplementary/S5_Fig.tif]

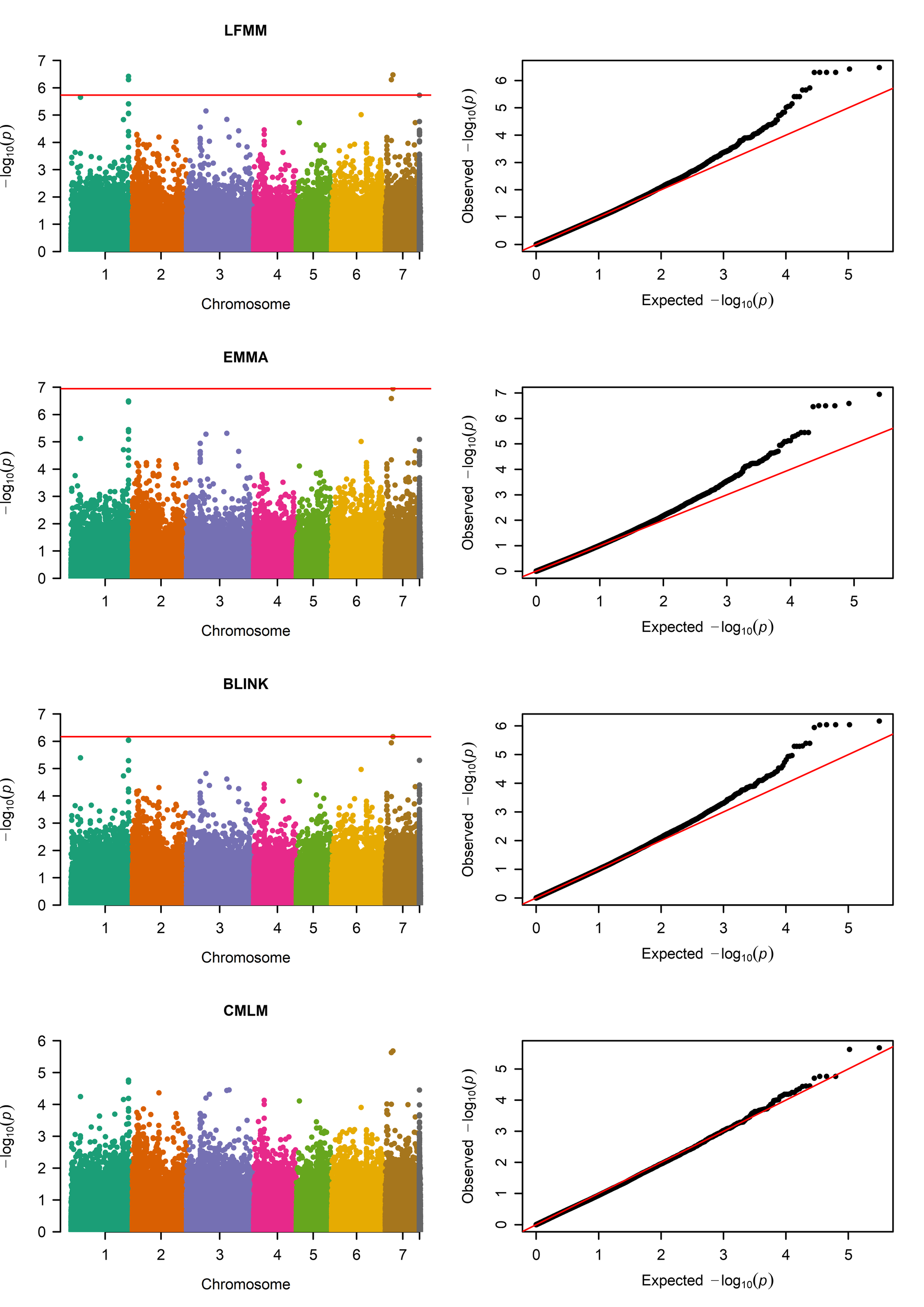

Supplement: S1 File — Field trial experimental design. The design follows a completely randomized block layout with four replicates. Each replicate consists of 10 sub-blocks, each containing 16 plots. Each plot (detail shown in the top right) is planted with three rows of 10 plants of the same genotype, with a spacing of 0.9 m between rows and 0.3 m between plants. The red dots indicate locations where soil samples were collected at 4 different depths (0–140 cm). Leaf samples, soil analyses, and root traits were also studied (illustrations shown at the bottom right). The blue icons represent irrigation pumps. S2 Fig. Correlation plot for soil ion content in 2021 (A) and 2022 (B) field sites. Heatmap representing Pearson’s correlation coefficients between soil ion concentrations measured at four different depths (0–140 cm). Color gradients indicate the Pearson’s correlation coefficient. Non-significant correlations at a p-value threshold of 0.05 are indicated with a cross. S3 Fig. Boxplot representing variation in ion content in 2021 and 2022 in the PMIGAP panel. Ion content is represented as mg/kg. p-values from the Wilcoxon test are represented. S4 Fig. Correlation plot for leaf ion content of the PMIGAP panel measured during the experimental field study. Heatmap representing Pearson’s correlation coefficients between BLUEs of all accessions of the panel observed in 2021 (A) and 2022 (B). Color gradients indicate the Pearson’s correlation coefficient. Non-significant correlations at a p-value threshold of 0.05 are indicated with a cross. S5 Fig. Correlation plot for ion content, root (A) and agro-morphological (B) traits in 2021 and 2022. Heatmap representing Pearson’s correlation coefficients between ion content, root and agro-morphological traits. Color gradients indicate the Pearson’s correlation coefficient. Significant correlations at a p-value threshold of 0.05 are indicated in bold. Roots traits are number of metaxylem vessels (MX_Number), mean area of metaxylem vessels (Meansize [file pone.0319140.s001.zip › Supplementary/S7_Fig.tif]

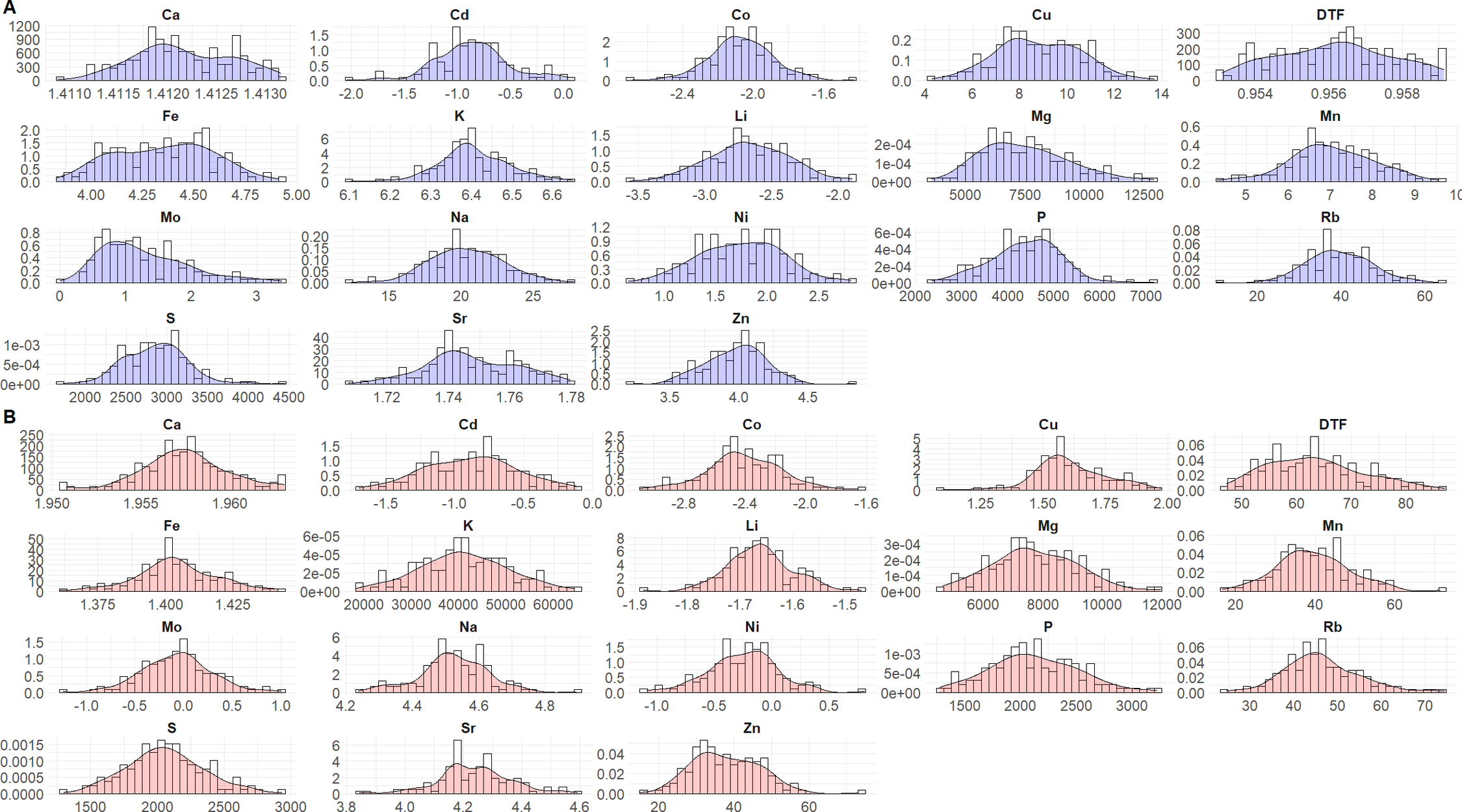

Supplement: S1 File — Field trial experimental design. The design follows a completely randomized block layout with four replicates. Each replicate consists of 10 sub-blocks, each containing 16 plots. Each plot (detail shown in the top right) is planted with three rows of 10 plants of the same genotype, with a spacing of 0.9 m between rows and 0.3 m between plants. The red dots indicate locations where soil samples were collected at 4 different depths (0–140 cm). Leaf samples, soil analyses, and root traits were also studied (illustrations shown at the bottom right). The blue icons represent irrigation pumps. S2 Fig. Correlation plot for soil ion content in 2021 (A) and 2022 (B) field sites. Heatmap representing Pearson’s correlation coefficients between soil ion concentrations measured at four different depths (0–140 cm). Color gradients indicate the Pearson’s correlation coefficient. Non-significant correlations at a p-value threshold of 0.05 are indicated with a cross. S3 Fig. Boxplot representing variation in ion content in 2021 and 2022 in the PMIGAP panel. Ion content is represented as mg/kg. p-values from the Wilcoxon test are represented. S4 Fig. Correlation plot for leaf ion content of the PMIGAP panel measured during the experimental field study. Heatmap representing Pearson’s correlation coefficients between BLUEs of all accessions of the panel observed in 2021 (A) and 2022 (B). Color gradients indicate the Pearson’s correlation coefficient. Non-significant correlations at a p-value threshold of 0.05 are indicated with a cross. S5 Fig. Correlation plot for ion content, root (A) and agro-morphological (B) traits in 2021 and 2022. Heatmap representing Pearson’s correlation coefficients between ion content, root and agro-morphological traits. Color gradients indicate the Pearson’s correlation coefficient. Significant correlations at a p-value threshold of 0.05 are indicated in bold. Roots traits are number of metaxylem vessels (MX_Number), mean area of metaxylem vessels (Meansize [file pone.0319140.s001.zip › Supplementary/S6_Fig.tif]

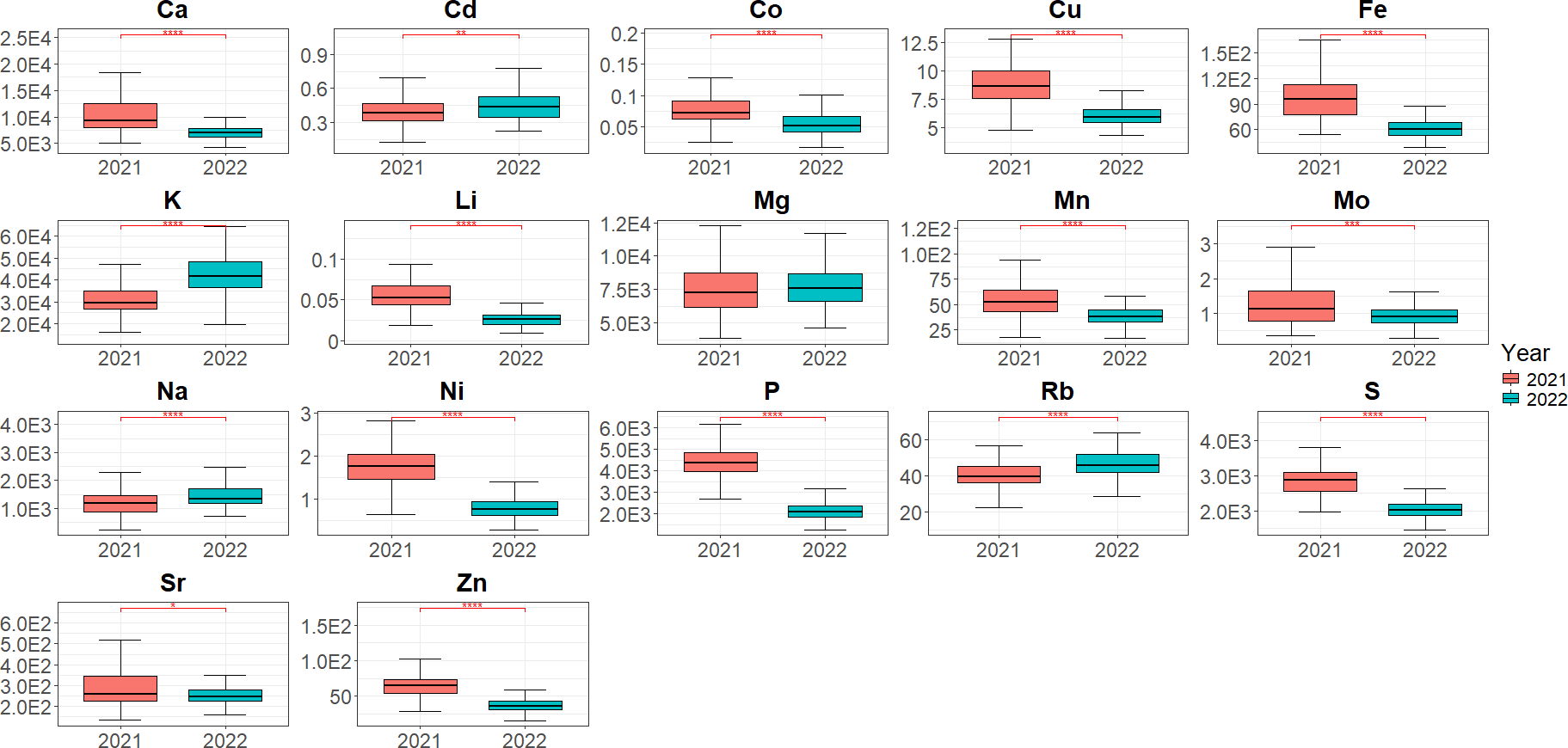

Supplement: S1 File — Field trial experimental design. The design follows a completely randomized block layout with four replicates. Each replicate consists of 10 sub-blocks, each containing 16 plots. Each plot (detail shown in the top right) is planted with three rows of 10 plants of the same genotype, with a spacing of 0.9 m between rows and 0.3 m between plants. The red dots indicate locations where soil samples were collected at 4 different depths (0–140 cm). Leaf samples, soil analyses, and root traits were also studied (illustrations shown at the bottom right). The blue icons represent irrigation pumps. S2 Fig. Correlation plot for soil ion content in 2021 (A) and 2022 (B) field sites. Heatmap representing Pearson’s correlation coefficients between soil ion concentrations measured at four different depths (0–140 cm). Color gradients indicate the Pearson’s correlation coefficient. Non-significant correlations at a p-value threshold of 0.05 are indicated with a cross. S3 Fig. Boxplot representing variation in ion content in 2021 and 2022 in the PMIGAP panel. Ion content is represented as mg/kg. p-values from the Wilcoxon test are represented. S4 Fig. Correlation plot for leaf ion content of the PMIGAP panel measured during the experimental field study. Heatmap representing Pearson’s correlation coefficients between BLUEs of all accessions of the panel observed in 2021 (A) and 2022 (B). Color gradients indicate the Pearson’s correlation coefficient. Non-significant correlations at a p-value threshold of 0.05 are indicated with a cross. S5 Fig. Correlation plot for ion content, root (A) and agro-morphological (B) traits in 2021 and 2022. Heatmap representing Pearson’s correlation coefficients between ion content, root and agro-morphological traits. Color gradients indicate the Pearson’s correlation coefficient. Significant correlations at a p-value threshold of 0.05 are indicated in bold. Roots traits are number of metaxylem vessels (MX_Number), mean area of metaxylem vessels (Meansize [file pone.0319140.s001.zip › Supplementary/S3_Fig.tif]

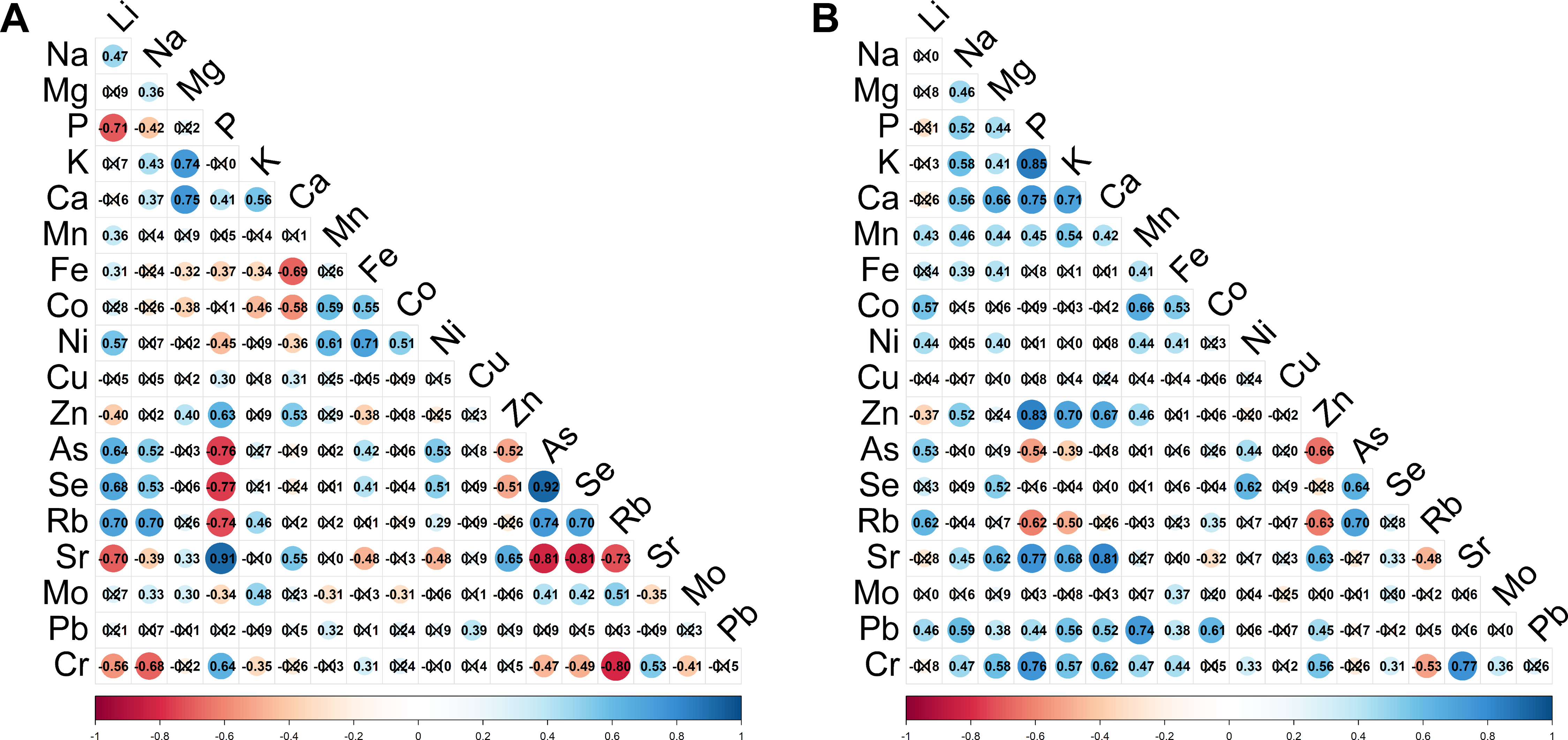

Supplement: S1 File — Field trial experimental design. The design follows a completely randomized block layout with four replicates. Each replicate consists of 10 sub-blocks, each containing 16 plots. Each plot (detail shown in the top right) is planted with three rows of 10 plants of the same genotype, with a spacing of 0.9 m between rows and 0.3 m between plants. The red dots indicate locations where soil samples were collected at 4 different depths (0–140 cm). Leaf samples, soil analyses, and root traits were also studied (illustrations shown at the bottom right). The blue icons represent irrigation pumps. S2 Fig. Correlation plot for soil ion content in 2021 (A) and 2022 (B) field sites. Heatmap representing Pearson’s correlation coefficients between soil ion concentrations measured at four different depths (0–140 cm). Color gradients indicate the Pearson’s correlation coefficient. Non-significant correlations at a p-value threshold of 0.05 are indicated with a cross. S3 Fig. Boxplot representing variation in ion content in 2021 and 2022 in the PMIGAP panel. Ion content is represented as mg/kg. p-values from the Wilcoxon test are represented. S4 Fig. Correlation plot for leaf ion content of the PMIGAP panel measured during the experimental field study. Heatmap representing Pearson’s correlation coefficients between BLUEs of all accessions of the panel observed in 2021 (A) and 2022 (B). Color gradients indicate the Pearson’s correlation coefficient. Non-significant correlations at a p-value threshold of 0.05 are indicated with a cross. S5 Fig. Correlation plot for ion content, root (A) and agro-morphological (B) traits in 2021 and 2022. Heatmap representing Pearson’s correlation coefficients between ion content, root and agro-morphological traits. Color gradients indicate the Pearson’s correlation coefficient. Significant correlations at a p-value threshold of 0.05 are indicated in bold. Roots traits are number of metaxylem vessels (MX_Number), mean area of metaxylem vessels (Meansize [file pone.0319140.s001.zip › Supplementary/S2_Fig.tif]

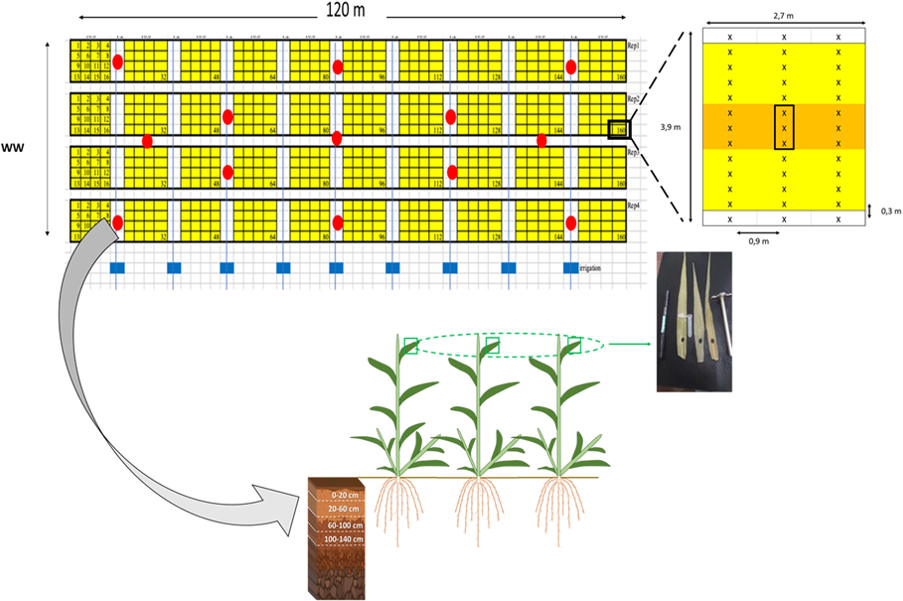

Supplement: S1 File — Field trial experimental design. The design follows a completely randomized block layout with four replicates. Each replicate consists of 10 sub-blocks, each containing 16 plots. Each plot (detail shown in the top right) is planted with three rows of 10 plants of the same genotype, with a spacing of 0.9 m between rows and 0.3 m between plants. The red dots indicate locations where soil samples were collected at 4 different depths (0–140 cm). Leaf samples, soil analyses, and root traits were also studied (illustrations shown at the bottom right). The blue icons represent irrigation pumps. S2 Fig. Correlation plot for soil ion content in 2021 (A) and 2022 (B) field sites. Heatmap representing Pearson’s correlation coefficients between soil ion concentrations measured at four different depths (0–140 cm). Color gradients indicate the Pearson’s correlation coefficient. Non-significant correlations at a p-value threshold of 0.05 are indicated with a cross. S3 Fig. Boxplot representing variation in ion content in 2021 and 2022 in the PMIGAP panel. Ion content is represented as mg/kg. p-values from the Wilcoxon test are represented. S4 Fig. Correlation plot for leaf ion content of the PMIGAP panel measured during the experimental field study. Heatmap representing Pearson’s correlation coefficients between BLUEs of all accessions of the panel observed in 2021 (A) and 2022 (B). Color gradients indicate the Pearson’s correlation coefficient. Non-significant correlations at a p-value threshold of 0.05 are indicated with a cross. S5 Fig. Correlation plot for ion content, root (A) and agro-morphological (B) traits in 2021 and 2022. Heatmap representing Pearson’s correlation coefficients between ion content, root and agro-morphological traits. Color gradients indicate the Pearson’s correlation coefficient. Significant correlations at a p-value threshold of 0.05 are indicated in bold. Roots traits are number of metaxylem vessels (MX_Number), mean area of metaxylem vessels (Meansize [file pone.0319140.s001.zip › Supplementary/S1_Fig.tif]
